# Privacy-preserving adaptive arrhythmia monitoring with wearable ECG, smartphone PPG, and federated on-device learning

**DOI:** 10.3389/fdgth.2026.1900115

**Published:** 2026-07-29

**Authors:** Aneta Kartali, Marija Koshtrevska, Stevan Jokić, Nenad Gligorić, Octavian M. Machidon

**Affiliations:** 1 Faculty of Computer and Information Science, University of Ljubljana, Ljubljana, Slovenia; 2 Faculty of Information Technology, Alfa BK University, Belgrade, Serbia; 3 Zentrix Lab LLC, Pančevo, Serbia

**Keywords:** arrhythmia monitoring, federated learning, mobile health, on-device AI, PPG, privacy-preserving healthcare, self-supervised learning, wearable ECG

## Abstract

**Introduction:**

Wearable electrocardiography (ECG) and smartphone technology create new opportunities for arrhythmia monitoring outside clinical environments. However, deployable mobile cardiac systems must address more than just classification accuracy. They must reliably acquire physiological signals under real-world conditions, protect the privacy of sensitive cardiac data, adapt to user- and sensor-specific variability, and minimize false alarms in daily use. We present Cardio-FL, a smartphone-based digital health prototype for privacy-preserving, adaptive arrhythmia monitoring.

**Methods:**

The system integrates wearable ECG acquisition with activity-aware on-device classification, camera-based photoplethysmography (PPG) pulse-regularity assessment, and federated on-device learning. ECG signals are streamed from a Movesense wearable sensor to the developed Android application via Bluetooth Low Energy, preprocessed locally, and converted into compact representations composed of derivative ECG samples and normalized RR-interval features. The deployed neural network follows an encoder-classifier-decoder architecture with only 5,575 parameters. The classifier distinguishes between normal beats, supraventricular ectopic beats (SVEB), and ventricular ectopic beats (VEB). At the same time, the encoder and decoder are further adapted on-device using unlabeled ECG segments and a reconstruction-based federated learning strategy. To improve signal representations without increasing runtime complexity, the model is pretrained prior to deployment. The encoder is first pretrained using masked reconstruction on the PTB-XL dataset and subsequently fine-tuned together with the classifier on the MIT-BIH beat-classification task before deployment. The developed Android application incorporates continuous ECG acquisition and preprocessing, on-device inference, and Flower-based federated encoder-decoder adaptation. Both inference and training are gated by user activity, while PPG-based pulse-regularity assessment is also performed after ECG alerts as a post-alert support workflow rather than as a clinically validated diagnostic confirmation step.

**Results:**

In offline evaluation, masked pretraining combined with validation-selected SVEB-aware calibration improves the deployed model performance from 92.80% to 94.15% accuracy and from 69.24% to 74.53% macro-F1 compared with MIT-BIH-only training. SVEB F1 improved from 35.86% to 46.11%, while VEB F1 increased from 75.44% to 80.31%.

**Discussion:**

The results demonstrate the feasibility of integrating wearable ECG acquisition, compact on-device inference, activity-aware operation, smartphone-camera PPG assessment, and reconstruction-based federated adaptation within a single Android application. Cardio-FL provides a practical framework for privacy-preserving and adaptive mobile arrhythmia monitoring. We further identify the validation steps required for real-world clinical integration.

## 1 Introduction

Cardiac rhythm monitoring is shifting from episodic clinical assessment to continuous, user-controlled sensing in everyday environments. Electrocardiography (ECG) remains the reference modality for rhythm interpretation because it directly measures the heart’s electrical activity and enables beat-level analysis of waveform morphology and timing. However, conventional clinical ECG and Holter monitoring workflows are not designed for unobtrusive, frequent, long-term use outside clinical settings. Wearable sensors, on the other hand, combined with smartphone technology, can help bridge this gap by enabling cardiac monitoring in daily life. At the same time, mobile deployment introduces new challenges. Physiological signals are acquired under uncontrolled conditions, mobile devices have limited computational and energy resources, users exhibit substantial physiological variability, and sensitive biomedical data should not be unnecessarily transferred to centralized servers.

Most arrhythmia detection studies either evaluate classification performance on public ECG benchmarks, such as the MIT-BIH dataset, or focus specifically on atrial fibrillation detection using photoplethysmography (PPG) (1, 2). While these studies are essential for reproducible model development, they do not fully address the deployment problem (3). A practical mobile monitoring system must support the complete sensing pipeline, including signal acquisition, preprocessing, inference, user feedback, and model adaptation. It must also determine when measurements are unreliable, manage motion-contaminated signals, adapt to user-specific characteristics without extensive clinician labeling, and preserve privacy during long-term use.

Recent portable ECG systems have demonstrated the feasibility of combining wearable acquisition hardware with embedded or smartphone-based machine-learning inference for arrhythmia detection (4, 5). However, many existing systems still rely on a static deployment paradigm in which a classifier is trained offline on curated datasets and then exported unchanged to a mobile device. This approach is limited in real-world settings because ECG morphology and rhythm characteristics vary across users, electrode positions, sensor properties, skin contact conditions, and daily activities. As a result, models that perform well under benchmark evaluation may generalize poorly during long-term personal monitoring.

PPG-based monitoring offers a complementary perspective. PPG signals can be acquired using smartwatches, pulse oximeters, or smartphone cameras, and deep-learning approaches have shown promising results for atrial fibrillation screening from pulse waveforms (6–8). Nevertheless, PPG measures peripheral blood-volume changes rather than cardiac electrical activity and is therefore more indirect than ECG. PPG recordings are also highly sensitive to motion artifacts, illumination changes, contact pressure, and peripheral perfusion. Rather than replacing ECG, PPG can therefore be used as a secondary post-alert assessment of pulse regularity and signal usability following the detection of an ECG anomaly.

Privacy is central to mobile cardiac monitoring. ECG and PPG recordings contain sensitive physiological information, and large-scale centralized data collection raises ethical, regulatory, and user-trust concerns. Federated learning partially addresses these issues by enabling distributed model training while keeping raw physiological data on local devices and sharing only model updates (9). Frameworks such as Flower have further improved the accessibility of federated-learning experimentation and deployment (10). Recent studies have applied federated learning to ECG-based arrhythmia detection and anomaly monitoring, including supervised classification of atrial fibrillation and unsupervised single-lead ECG anomaly detection across devices (11, 12). However, many of these approaches remain benchmark- or simulation-oriented and do not integrate federated learning into a complete smartphone-based sensing workflow that includes real-time acquisition, local signal processing, user-facing alerts, and multimodal confirmation.

Another practical limitation is the scarcity of labeled data during ordinary use. ECG recordings collected in daily life are rarely accompanied by clinician-verified annotations, making fully supervised on-device model personalization unfeasible. Self-supervised and unsupervised learning strategies are therefore attractive because they can adapt signal representations using unlabeled physiological data. Reconstruction-based learning is particularly well-suited to conservative mobile adaptation: the encoder can learn user- and sensor-specific morphology from local ECG windows, while the arrhythmia-classification layer remains fixed. This separation reduces the risk of noisy or unlabeled user data directly altering the decision boundary while still enabling privacy-preserving personalization.

In this work, we present Cardio-FL, a smartphone-centered prototype for privacy-preserving and adaptive arrhythmia monitoring. The system combines wearable ECG sensing with on-device beat classification, smartphone-camera PPG pulse-regularity assessment, and federated reconstruction learning within a unified mobile-health workflow. ECG signals are streamed from a Movesense wearable sensor to an Android application over Bluetooth Low Energy, preprocessed locally, and segmented into compact beat representations combining ECG morphology and RR-interval context. The system then classifies beats into normal, supraventricular ectopic beat (SVEB), and ventricular ectopic beat (VEB) categories. When an ECG anomaly is detected, the application prompts the user to record a short PPG segment using the smartphone camera. The PPG module evaluates signal usability and pulse regularity as a secondary confirmation step rather than as an independent diagnostic classifier. Furthermore, unlabeled ECG windows collected during operation are used to update the encoder and decoder through federated reconstruction learning, thus enabling privacy-preserving adaptation to user- and sensor-specific signal characteristics during system use.

To support efficient mobile deployment, the proposed model uses a compact encoder-classifier-decoder architecture with only 5,575 parameters and a 68-dimensional ECG/RR feature representation. The classifier is trained offline and remains fixed during deployment, while the encoder and decoder are adapted locally using reconstruction-based federated learning on unlabeled ECG windows. Prior to deployment, the encoder is pretrained using masked reconstruction on PTB-XL lead-II ECG windows and subsequently fine-tuned together with the classifier on the MIT-BIH beat-classification task. This training strategy separates representation learning from supervised arrhythmia classification: PTB-XL provides large-scale unlabeled morphological diversity, while MIT-BIH supplies expert beat annotations for the deployed normal/SVEB/VEB classification task (13–15).

The primary contribution of this work is not a standalone arrhythmia classifier but an integrated mobile health architecture for adaptive and privacy-preserving cardiac monitoring. Specifically, this paper contributes:

- **A deployable wearable-to-smartphone arrhythmia-monitoring pipeline** integrating ECG acquisition, on-device preprocessing, compact beat representation, and local arrhythmia classification.
- **A privacy-preserving adaptation strategy** based on reconstruction-driven local and federated learning of unlabeled ECG windows, enabling personalization without transferring raw physiological data or requiring clinician annotations during daily use.
- **A complete mobile monitoring workflow** that combines pretrained lightweight ECG classification, activity-aware gating, and smartphone-camera PPG pulse-regularity assessment within a practical Android deployment framework.

The remainder of this paper is organized as follows. Section 2 reviews related work on wearable ECG monitoring, PPG-based arrhythmia screening, federated learning for cardiac monitoring, and on-device personalization. Section 3 describes the proposed system architecture, signal-processing pipelines, datasets, and the federated learning workflow. Section 4 presents offline evaluation results and the Android implementation. Section 5 discusses limitations, deployment considerations, and remaining validation requirements for clinical integration. Finally, Section 6 concludes the paper.

## 2 Background and related work

The growing availability of wearable sensors and smartphones has enabled a shift from episodic cardiac assessment toward continuous monitoring in everyday environments. Two sensing modalities dominate this space: electrocardiography (ECG) and photoplethysmography (PPG). ECG remains the clinical reference for rhythm analysis because it directly measures the heart’s electrical activity and enables beat-level assessment of waveform morphology and timing. PPG, in contrast, measures peripheral blood volume changes and can be conveniently acquired via smartwatches, pulse oximeters, and smartphone cameras. Although PPG is easier to collect continuously, it provides a more indirect representation of cardiac activity and is more susceptible to motion, contact, illumination, and perfusion artifacts. Consequently, ECG and PPG are best viewed as complementary modalities: ECG provides the primary basis for arrhythmia assessment, while PPG can support low-burden screening and confirmation of pulse irregularities (6–8).

### 2.1 Wearable and smartphone ECG systems

Wearable ECG systems have become an important approach for ambulatory arrhythmia monitoring by enabling measurements outside clinical environments while reducing the burden associated with traditional Holter monitoring. Public datasets such as MIT-BIH and PTB-XL have played a central role in this development by providing standardized benchmarks for algorithm evaluation and benchmarking (13–15). MIT-BIH remains one of the most widely used resources for beat-level arrhythmia classification, including normal, supraventricular ectopic, and ventricular ectopic beats, whereas PTB-XL provides a large-scale corpus suitable for pretraining and self-supervised representation learning (14, 15). Classical signal-processing techniques, including QRS detection and beat segmentation, continue to form the foundation of many ECG-analysis pipelines, with the Pan-Tompkins algorithm remaining a widely adopted solution for real-time R-peak detection (16).

Recent portable ECG solutions combine wearable acquisition hardware with embedded or smartphone-based machine-learning models capable of real-time arrhythmia detection. These studies demonstrate that reliable ECG analysis can be performed on resource-constrained devices (4, 5). However, most systems still rely on static deployment strategies in which models are trained centrally and subsequently deployed without further adaptation. This remains a significant limitation because ECG morphology varies across users, recording conditions, sensor configurations, and daily activities. As a result, strong benchmark performance does not necessarily translate into robust long-term monitoring in real-world settings.

Recent machine-learning studies further illustrate the breadth of physiological-signal modeling relevant to this area, including hybrid ECG arrhythmia classifiers, combined ECG and chest-X-ray heart-disease detection, wearable ECG and bioimpedance classification, and ensemble learning for wearable physiological signals (17–20).

### 2.2 PPG-based atrial fibrillation detection

PPG has received considerable attention for atrial fibrillation (AF) screening because it can be acquired continuously using widely available consumer devices, including smartwatches and smartphones. Early approaches relied on handcrafted pulse-interval features and rhythm-irregularity measures, whereas recent studies have increasingly adopted deep-learning models trained directly on PPG waveforms (6–8). The increasing availability of large-scale real-world smartphone PPG datasets further supports the development of mobile cardiovascular monitoring applications and data-driven assessment methods (21). These studies have demonstrated that AF can be detected from PPG recordings in both clinical and ambulatory settings, highlighting the potential of PPG for large-scale and low-burden rhythm monitoring (6, 7).

Despite these advances, PPG should not be regarded as a direct replacement for ECG. Unlike ECG, which measures cardiac electrical activity, PPG captures the peripheral hemodynamic response and is therefore more susceptible to motion artifacts, contact variability, illumination changes, and perfusion-related effects. Recent multimodal approaches have consequently explored the complementary use of ECG and PPG. For example, SiamAF leverages paired ECG and PPG recordings to learn shared representations that support AF detection from either modality (22). These studies reinforce the view that ECG and PPG provide complementary information rather than interchangeable measurements. Accordingly, PPG is increasingly being considered as a supporting modality for rhythm assessment rather than a standalone replacement for ECG-based monitoring.

### 2.3 Federated and privacy-preserving learning for mobile cardiac monitoring

The privacy-sensitive nature of physiological data presents a major challenge for mobile cardiac monitoring. Traditional centralized machine-learning pipelines typically require physiological recordings to be collected or uploaded to a central server for training and, in some cases, inference. Federated learning addresses this issue by enabling collaborative model training across distributed devices while keeping raw recordings on-device and sharing only model updates with a central server (9). Frameworks such as Flower have further accelerated the development and deployment of federated-learning systems in heterogeneous real-world environments (10). Nevertheless, federated learning does not eliminate all privacy concerns, as model updates may still reveal information under certain threat models, motivating complementary techniques such as secure aggregation and differential privacy (23, 24).

Recent studies have applied federated learning to ECG-based arrhythmia detection and anomaly monitoring. Alreshidi et al. proposed Fed-CL, a federated framework for ECG-based atrial fibrillation classification that emphasizes privacy-preserving collaborative learning and communication-efficient model exchange (11). Kapsecker and Jonas extended this direction to cross-device unsupervised ECG anomaly detection, combining federated learning with on-device personalization in a mobile setting (12). Their work is particularly relevant because it highlights the practical challenges of heterogeneous mobile environments, including privacy preservation, inter-subject variability, and the scarcity of labeled data.

The limited availability of annotated ECG recordings during everyday use remains a major obstacle to personalized mobile monitoring. Since clinician-verified labels are rarely available outside controlled studies, recent research has increasingly explored self-supervised and unsupervised learning strategies. Reconstruction-based objectives, contrastive learning, and masked-signal modeling enable representation learning from unlabeled physiological recordings while reducing dependence on manual annotation. These approaches offer a promising direction for privacy-preserving personalization in long-term mobile monitoring scenarios.

### 2.4 On-device inference, personalization, and deployment gaps

On-device inference plays a central role in mobile-health applications by reducing latency, preserving privacy, and enabling operation without continuous cloud connectivity. However, practical deployment imposes constraints that go beyond classification performance. Models must be sufficiently lightweight to support real-time inference and, where applicable, local training without excessive memory, energy, thermal, or communication overhead.

Although many studies report classification metrics such as accuracy, F1-score, sensitivity, specificity, or AUC, fewer evaluate deployment-oriented runtime properties, including inference latency, battery consumption, communication costs, or robustness to mobile sensing conditions. This gap remains an important challenge for digital-health systems, where successful offline benchmark performance does not necessarily translate into reliable operation on resource-constrained devices.

Personalization represents a related challenge. ECG morphology and rhythm characteristics vary substantially across individuals, while data collected across mobile devices are inherently non-identically distributed. Federated learning can help create shared representations across users, but effective long-term monitoring often requires mechanisms that adapt to user-specific signal characteristics. Recent work has shown that combining federated learning with local adaptation can improve performance in heterogeneous mobile environments (12), emphasizing the importance of personalization in future wearable cardiac-monitoring systems.

### 2.5 Positioning of the present work

Existing research has made significant progress in three complementary areas: ECG-based arrhythmia classification, PPG-based rhythm screening, and federated learning for privacy-preserving physiological modeling. However, these directions are often investigated independently. Fewer studies integrate wearable ECG acquisition, smartphone-based inference, multimodal confirmation, and federated adaptation into a single, deployable mobile health system.

The present work is positioned at this intersection. Rather than proposing a new standalone arrhythmia classification model, we focus on integrating wearable sensing, on-device inference, PPG-assisted confirmation, and privacy-preserving adaptation into a unified smartphone-centered architecture. In doing so, we aim to bridge the gap between benchmark-oriented algorithm development and practical mobile deployment for adaptive arrhythmia monitoring.

## 3 Materials and methods

### 3.1 System design and runtime workflow

Cardio-FL is a smartphone-based cardiac-monitoring system that combines wearable ECG acquisition, on-device beat classification, federated model adaptation, activity-aware operation, and smartphone-camera PPG pulse-regularity assessment. The system was developed to evaluate how lightweight arrhythmia detection and privacy-preserving personalization can be integrated into a practical Android application while keeping raw physiological data on the user’s device. In the present prototype, smartphone-camera PPG is used as a post-alert signal-usability and pulse-regularity assessment workflow rather than as an independent diagnostic validator of individual ECG beat classifications.

The runtime workflow consists of five main components: wearable ECG acquisition, ECG signal processing, beat classification, federated learning, and PPG pulse assessment. The Android application coordinates these components. ECG signals are streamed from a Movesense wearable sensor over Bluetooth Low Energy (BLE), buffered and preprocessed on the smartphone, and converted into beat-level ECG/RR representations for classification. When the user is stationary, the application performs on-device inference and stores unlabeled ECG segments for later use in local and federated model adaptation.

The application provides real-time feedback on sensor connectivity, signal acquisition, activity state, ECG processing, classification results, PPG status, and system notifications. This allows users to verify that the wearable sensor is connected and that physiological data are being correctly acquired. When the classifier detects an abnormal beat, the application notifies the user and requests a short camera-based PPG recording. Users may also initiate a PPG recording manually through the application interface.

**Figure 1:**
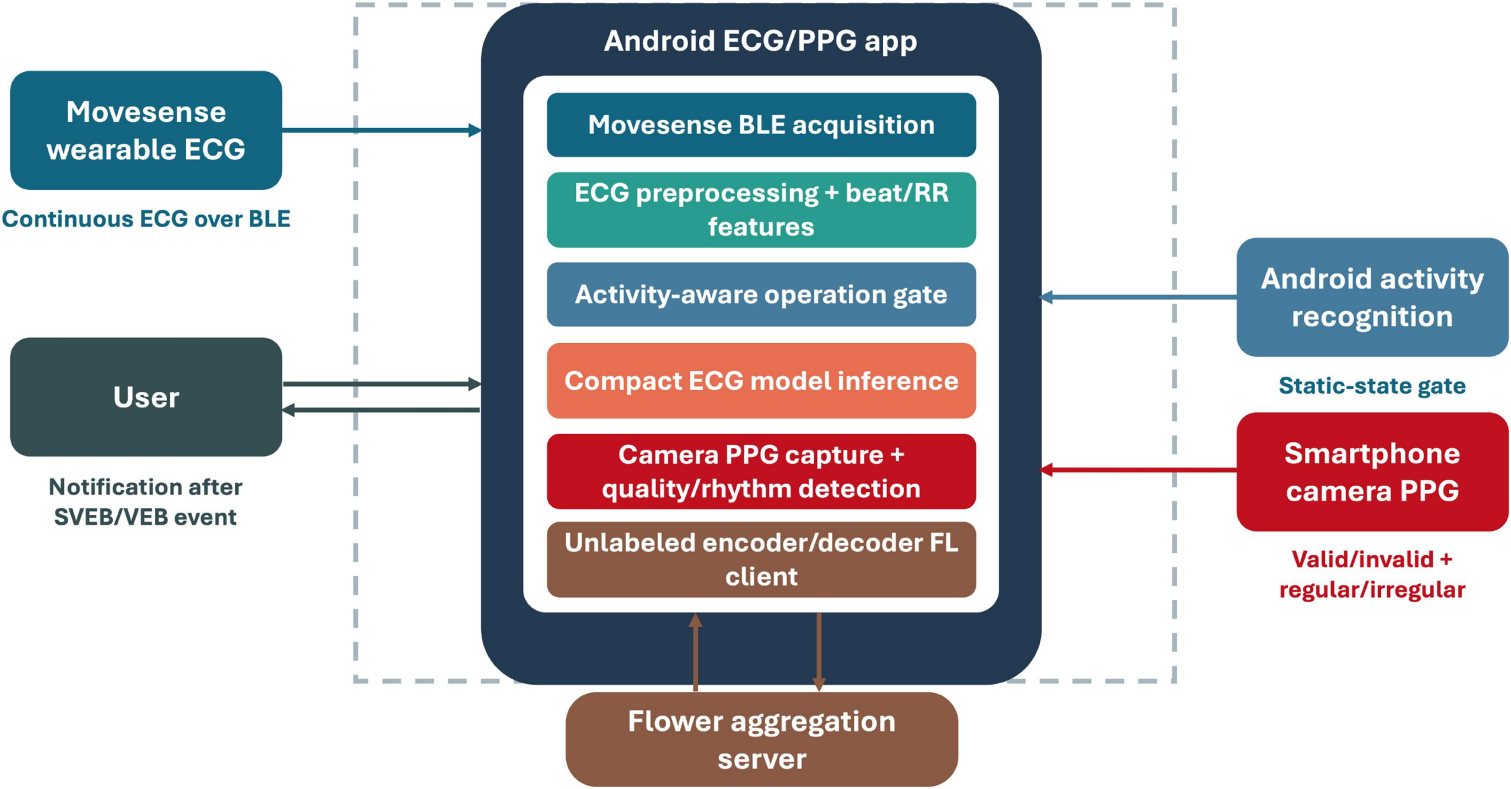
Overview of the Cardio-FL system. ECG signals are streamed from a wearable sensor to an Android application, where user activity recognition, signal processing, ECG beat classification, and federated learning are performed. When an abnormal ECG pattern is detected, the user is prompted to record a smartphone-camera PPG signal for pulse-regularity assessment. Raw ECG and PPG recordings remain on the smartphone, while only trainable model parameters are exchanged with the federated server.

**Figure 2:**
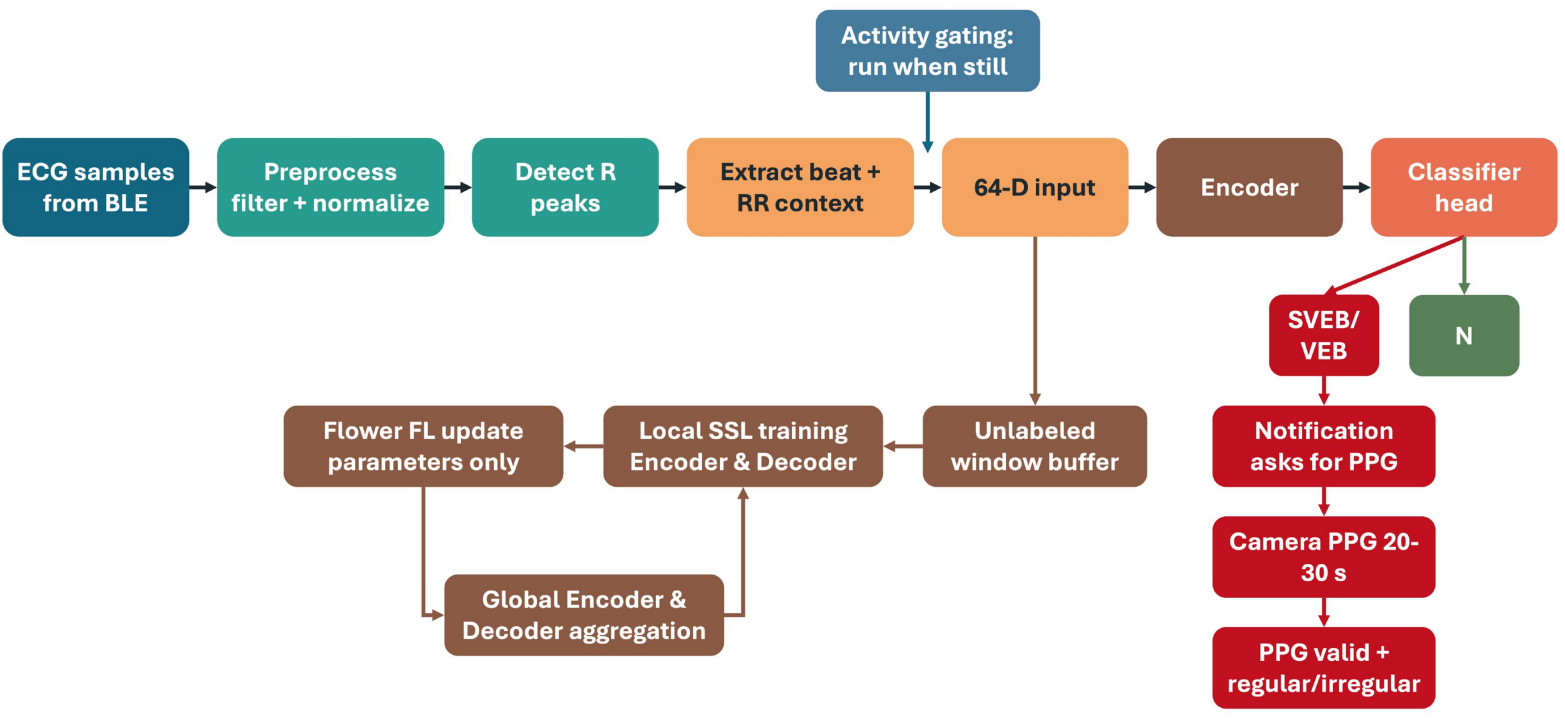
Runtime monitoring and adaptation workflow. ECG signals acquired from the wearable sensor are preprocessed and converted into beat-level ECG/RR representations for on-device classification. Activity-aware gating controls both inference and model adaptation. Detected anomalies trigger an optional PPG assessment, while unlabeled ECG segments are stored for reconstruction-based federated learning.

**Figure 3:**
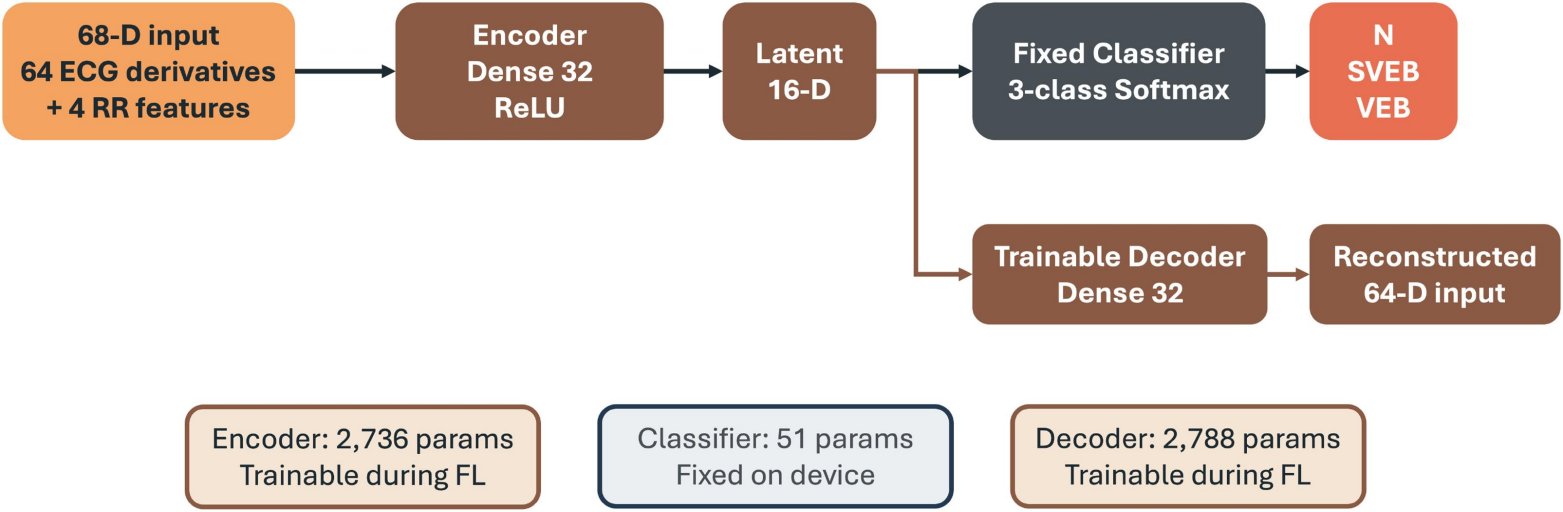
Encoder-classifier-decoder architecture used for on-device ECG analysis. The classifier predicts normal beats, supraventricular ectopic beats (SVEB), and ventricular ectopic beats (VEB). During federated adaptation, only the encoder and decoder are updated through a reconstruction objective, while the classifier remains fixed.

**Figure 4:**
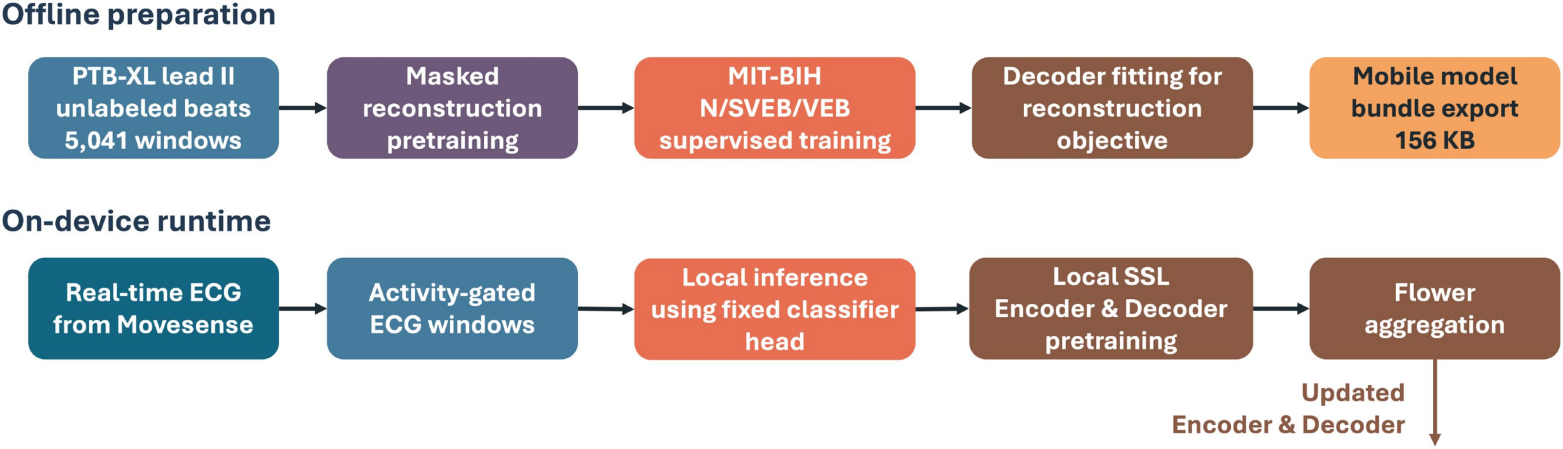
Model training and deployment workflow. The encoder is first pretrained using masked reconstruction on PTB-XL lead-II ECG windows and subsequently fine-tuned with the classifier on the MIT-BIH beat-classification task. The resulting model is deployed on Android, where it supports on-device inference and federated masked reconstruction learning using unlabeled ECG data collected during operation.

The overall workflow is illustrated in Figures 1, 2. Figure 1 shows the distribution of processing and data storage between the smartphone and the federated server. Raw ECG and PPG recordings remain on the smartphone, while only encoder and decoder model parameters are exchanged during federated training. Figure 2 summarizes the runtime sequence, including ECG acquisition, beat extraction, on-device classification, optional PPG pulse assessment, and federated model updates. Figure 3 presents the model architecture, and Figure 4 illustrates the relationship between offline pretraining, supervised training, mobile deployment, and on-device adaptation.

The present implementation builds on a commercial wearable ECG sensor and focuses on the smartphone-side monitoring workflow. Accordingly, this work does not propose new ECG acquisition hardware, electrode configurations, or analog front-end electronics. Instead, it investigates the integration of wearable ECG sensing, on-device inference, PPG-based pulse assessment, and privacy-preserving model adaptation within a mobile monitoring system.

### 3.2 ECG acquisition, preprocessing, and beat representation

ECG signals are acquired from a Movesense wearable sensor using the Movesense Android software development kit (25). The Android application scans for available sensors, establishes a BLE connection, subscribes to the ECG stream, and receives ECG samples continuously while recording is enabled. All signal processing is performed locally on the smartphone, and ECG recordings are not uploaded to external servers.

ECG preprocessing is implemented directly within the Android application and operates on ECG signals sampled at 128 Hz. The incoming signal is first corrected for baseline wander using a trimmed moving-average estimate computed over a 0.20 s window (27 samples) with a trim fraction of 0.25. The signal is then filtered using second-order high-pass (0.67 Hz) and low-pass (40 Hz) biquad filters with Q=0.7071, applied forward and backward to achieve zero-phase filtering. A 50 Hz notch filter (Q=30) is also applied. The filtered signal is smoothed with an 11-sample quadratic Savitzky–Golay filter and then standardized via z-score normalization computed from the current ECG buffer, with a variance floor of $10^{-6}$.

R peaks are detected using a threshold-based local-maximum detector with a refractory period of 0.25 s. The detection threshold is derived from the amplitude characteristics of the preprocessed signal and adapts to the current ECG buffer. The implementation follows the general principles of the Pan–Tompkins algorithm for real-time QRS detection based on signal conditioning, peak detection, and refractory-period enforcement (16). Beat-centered windows are then extracted around each detected R peak.

Each beat window contains 64 samples centered on the detected R peak. At the 128 Hz sampling rate, this corresponds to a 0.5 s segment consisting of approximately 32 samples before and 32 samples after the R peak. The model input is constructed from the first derivative of this preprocessed beat segment, together with four normalized RR-interval features that describe the local and longer-term rhythm context.

Each beat is therefore represented by a compact 68-dimensional vector,

$$x=\left[\Delta s_{1\;:\;64},r_{\mathrm{local}}^{-},r_{\mathrm{local}}^{+},r_{\mathrm{global}}^{-},r_{\mathrm{global}}^{+}\right]\in R^{68},$$

where the first 64 elements correspond to the derivative of a beat-centered ECG segment and the remaining four elements represent normalized preceding and following RR intervals. The derivative ECG features capture local waveform morphology, while the RR features provide short-term and longer-term rhythm context. This representation combines morphological and temporal information while maintaining a sufficiently small input size for mobile deployment.

The 68-dimensional ECG/RR representation was selected as a compact, mobile-oriented combination of beat morphology and rhythm context, adapted from prior MIT-BIH heartbeat-classification work that combines ECG morphology with normalized RR-interval features (1). The 64 derivative ECG samples preserve local beat-shape information around the detected R peak while reducing sensitivity to slow baseline variation. The four RR features encode normalized timing context: the preceding and following RR intervals are each divided by a local RR mean computed from up to the 80 most recent preceding RR intervals and by a global RR mean computed from up to the 400 most recent preceding RR intervals, with the preceding RR interval used as fallback when insufficient history is available. Compared with longer raw ECG windows, wavelet-based features, or spectrogram representations, this compact vector reduces memory and computation requirements and can be generated directly within the real-time Android preprocessing pipeline, making it suitable for on-device inference and federated adaptation.

Signal quality is managed through the preprocessing pipeline, R-peak validation, beat-window selection, and activity-aware gating. ECG segments that do not produce valid beat windows are excluded from both inference and model adaptation. The current implementation therefore performs signal-usability screening rather than a clinically validated ECG signal-quality-index calculation.

Inference and model adaptation are performed only when the user is stationary. To determine the user’s activity state, the current implementation uses the smartphone inertial sensing stack through a phone-side motion detector, with linear acceleration used when available and accelerometer fallback otherwise. ECG classification and federated learning updates are enabled only during static periods, thereby reducing the influence of motion-related artifacts on both arrhythmia detection and model adaptation.

### 3.3 Model architecture, datasets, and offline training

The deployed model employs an encoder-classifier-decoder architecture that supports both beat classification and reconstruction-based adaptation. Given a 68-dimensional input vector x, the encoder generates a compact latent representation

$$z=E_{\theta }(x),$$

where $E_{\theta }$ consists of a fully connected layer with 32 ReLU units followed by a 16-dimensional latent layer. The classifier

$$\hat{y}=C_{\phi }(z)$$

maps the latent representation z to three softmax classes: normal beat (N), supraventricular ectopic beat (SVEB), and ventricular ectopic beat (VEB). The decoder

$$\hat{x}=D_{\psi }(z)$$

reconstructs the original 68-dimensional input representation x from the latent vector z.

The classifier $C_{\phi }$ is trained offline using labeled ECG data and remains fixed during deployment. In contrast, the encoder $E_{\theta }$ and decoder $D_{\psi }$ are updated through local and federated reconstruction learning on unlabeled ECG segments collected during operation. This separation allows the latent representation to adapt to user- and sensor-specific signal characteristics without requiring beat-level annotations. The complete model structure is summarized in Table 1.

**Table 1:** Deployed android model structure and parameter exposure.

Component	Role	Number of parameters	On-device status
Input representation	64 derivative ECG samples and 4 normalized RR features	–	Input only
Encoder dense layer	Maps 68-dimensional ECG/RR vector into 32 hidden units	2,208	Trainable
Latent layer	Produces a 16-dimensional latent representation	528	Trainable
Classifier head	Maps latent representations to N/SVEB/VEB probabilities	51	Fixed
Decoder hidden layer	Maps latent representations to 32 reconstruction units	544	Trainable
Decoder output layer	68-dimensional reconstruction output	2,244	Trainable
Total model	Encoder, classifier, decoder	5,575	–
Federated subset	Encoder and decoder only	5,524	Trainable

The complete learning pipeline is therefore intentionally separated into supervised discrimination and unsupervised adaptation terms. Offline pretraining first estimates an initialization for the encoder $E_{\theta }$ and decoder $D_{\psi }$ by minimizing reconstruction loss on unlabeled windows of the PTB-XL dataset $D_{\mathrm{PTB}}$,

$$(\theta_{0},\psi_{0})=\arg\;\min_{\theta ,\psi }\frac{1}{\left|D_{\mathrm{PTB}}\right|}\sum_{x\in D_{\mathrm{PTB}}}L_{\mathrm{rec}}(x;\theta ,\psi ).$$

The supervised MIT-BIH stage then fine-tunes the encoder $E_{\theta }$ and learns the deployed classifier $C_{\phi }$ by minimizing beat-classification loss over labeled beats from the MIT-BIH dataset $D_{\mathrm{MIT}}$,

$$(\theta^{⋆},\phi^{⋆})=\arg\;\min_{\theta ,\phi }\frac{1}{\left|D_{\mathrm{MIT}}^{\mathrm{train}}\right|}\sum_{(x,y)\in D_{\mathrm{MIT}}^{\mathrm{train}}}L_{\sup}(x,y;\theta ,\phi ).$$

During deployment, classifier parameters $\phi^{⋆}$ are kept fixed, while local and federated adaptation update only encoder and decoder parameters (θ,ψ) using unlabeled reconstruction loss. Thus, reconstruction adaptation is not assumed to directly optimize arrhythmia classification; it is used as a conservative representation-adaptation mechanism whose classification effect must be evaluated empirically.

Two public ECG datasets were used during model development. The PTB-XL dataset was used for representation pretraining (15). The dataset contains 21,837 clinical 12-lead ECG recordings from 18,885 patients and provides substantially greater morphological diversity than MIT-BIH. Because the deployed task is beat-level classification, PTB-XL diagnostic labels were not used. Instead, lead-II recordings from a subset of 500 low-rate records were converted into 5,041 unlabeled beat windows represented using the 68-dimensional ECG/RR feature format.

The MIT-BIH Arrhythmia Database was used for supervised beat classification (13, 14). The dataset contains 48 half-hour two-channel ambulatory ECG recordings sampled at 360 Hz and annotated by clinical experts. For supervised training, the MLII channel was used when available, otherwise, the first available ECG channel was used. Beat annotations were mapped to the three deployed classes (N, SVEB, and VEB), and each beat was converted into the same 68-dimensional ECG/RR representation used by the Android application.

To avoid record-level leakage, the MIT-BIH evaluation used a strict record-disjoint train/validation/test split. This is reported as record-level separation because the evaluation can be verified from the public record identifiers, whereas complete subject-identity separation beyond the record metadata should not be overclaimed. The training split contained records 101, 106, 108, 109, 112, 114, 115, 116, 118, 119, 122, and 124; the validation split contained records 201, 203, 205, 207, 208, 209, 215, 220, 223, and 230; and the held-out test split contained records 100, 103, 105, 111, 113, 117, 121, 123, 200, 202, 210, 212, 213, 214, 219, 221, 222, 228, 231, 232, 233, and 234. No record was shared across splits. The corresponding N/SVEB/VEB beat counts were 23,908/149/1,234 for training, 21,916/794/2,554 for validation, and 44,218/1,836/3,219 for testing.

Offline training was performed in three stages. First, the encoder $E_{\theta }$ and decoder $D_{\psi }$ were pretrained using masked reconstruction on unlabeled PTB-XL beat windows. For each input x, a corrupted version $\tilde{x}$ was generated by masking 15% of input values and adding independent Gaussian noise ($\epsilon ∼N(0,0.01^{2})$) to all input dimensions. The encoder and decoder were then trained to reconstruct the original signal by minimizing the reconstruction objective,

$$L_{\mathrm{rec}}={\left\|x-D_{\psi }(E_{\theta }(\tilde{x}))\right\|}_{2}^{2}.$$

Second, the encoder $E_{\theta }$ and classifier $C_{\phi }$ were trained on labeled MIT-BIH beats using sparse categorical cross-entropy,

$$L_{\sup}=-\sum_{k=1}^{3}y_{k}\log\;{\hat{y}}_{k}.$$

where $\hat{y}=C_{\phi }(E_{\theta }(x))$. Finally, the decoder was fitted on MIT-BIH training windows while keeping the encoder fixed. This step produced a model containing both a classification pathway and a reconstruction pathway for subsequent local and federated adaptation. The complete training workflow is summarized in Table 2.

**Table 2:** ECG model training and deployment stages.

Stage	Data source	Objective	Output
Unlabeled pretraining	PTB-XL lead-II subset: 500 records, 5,041 beat	Masked reconstruction of 68-dimensional ECG/RR windows	Encoder initialization
Supervised training	MIT-BIH beat windows mapped to N/SVEB/VEB	Sparse categorical cross-entropy	Encoder and classifier head
Decoder fitting	MIT-BIH training windows	Reconstruction loss with fixed encoder	Decoder for on-device FL
Mobile deployment	Trained model weights and deployment metadata	Android deployment package	156 KB model
On-device adaptation	Real-time ECG windows from Movesense wearable	Reconstruction learning on unlabeled ECG segments	Federated encoder and decoder updates

To assess the robustness of the reported test-set results, confidence intervals were estimated using bootstrap resampling at the MIT-BIH record level. Resampling entire records rather than individual beats preserved the same independence assumption used in the train/validation/test split. For paired comparisons on the same test records, including SVEB-aware calibration and the no-adaptation/local-adaptation/federated-adaptation comparison, paired record-level bootstrap intervals were computed. To examine the contribution of the compact ECG/RR representation, we performed a post hoc feature-ablation analysis on the selected calibrated model. Either the four RR features or the 64 derivative ECG samples were replaced with their training-set mean values, and the unchanged classifier was reevaluated on the held-out test set. This allowed us to estimate the relative importance of rhythm-context features and local ECG morphology for the final beat-classification performance.

### 3.4 Federated on-device reconstruction learning

The Android application implements federated learning using the Flower framework (10). The same reconstruction objective used during offline pretraining is applied during on-device adaptation. Buffered ECG windows are normalized locally, corrupted by additive noise, and used to train the encoder and decoder to reconstruct the original signal. The classifier remains fixed throughout this process and is excluded from all local and federated updates. Local inference and reconstruction training are executed directly on the smartphone, while Flower coordinates the exchange and aggregation of trainable model parameters across participating clients.

During federated communication, only encoder and decoder weights are exchanged with the server. At the same time, raw ECG windows, PPG recordings, beat annotations, and other user-specific physiological data remain on the device. Because the implementation does not include secure aggregation, differential privacy, or a formal model-inversion/privacy-leakage analysis, the privacy claim is limited to avoiding raw physiological data transfer in the evaluated prototype. The trainable federated subset contains 5,524 parameters corresponding to the encoder and decoder. For evaluation purposes, the Flower server was configured with full client participation, 20 communication rounds, local batches of 32 ECG windows, and three local training epochs per round. During testing, the minimum number of participating clients was set to one to simplify deployment and debugging.

Federated aggregation follows the FedAvg procedure (9). If θ and ψ denote the encoder and decoder parameters, at communication round t, each client i receives the current global parameters $\left(\theta_{t},\psi_{t}\right)$, performs local reconstruction training on its ECG windows, and returns updated parameters $\left(\theta_{t}^{\left(i\right)},\psi_{t}^{\left(i\right)}\right)$. The server computes the next global model as

$$(\theta_{t+1},\psi_{t+1})=\sum_{i=1}^{K}\frac{n_{i}}{\sum_{j}n_{j}}(\theta_{t}^{\left(i\right)},\psi_{t}^{\left(i\right)}),$$

where $n_{i}$ denotes the number of training windows contributed by client i.

By restricting federated updates to the encoder and decoder, the system adapts the latent ECG representation using unlabeled data collected during operation while preserving the fixed classification layer obtained during supervised training. This allows the model to adapt to user- and device-specific ECG patterns using unlabeled data collected during daily use without changing the classifier trained on expert-annotated MIT-BIH data. As a result, the model can learn the characteristics of local ECG signals while reducing the likelihood that noisy, unlabeled data directly affect the final N/SVEB/VEB classification decisions.

To examine whether this reconstruction-based adaptation changes downstream beat-classification behavior, we additionally conducted a post hoc pseudo-client experiment on the held-out MIT-BIH test records. Each test record was treated as a simulated client, with the first half of the record used as unlabeled data for adaptation and the second half reserved for labeled evaluation. This setup allowed us to compare fixed inference without adaptation, independent local reconstruction adaptation, and federated reconstruction adaptation with client-weighted FedAvg aggregation across records.

### 3.5 PPG assessment and Android deployment

The Android application includes a smartphone-camera PPG module that can be triggered automatically after an abnormal ECG prediction or started manually by the user. When the ECG classifier detects an abnormal beat with sufficient confidence, the application displays a rate-limited notification requesting a short PPG recording. The PPG signal is extracted from the red-channel intensity recorded by the smartphone camera, following the principle that finger contact with the illuminated camera produces pulsatile optical changes associated with blood-volume variation (26). The PPG prompt is therefore used as a post-alert pulse-assessment step, not as confirmation of the specific ECG beat classification, because smartphone-camera PPG reflects peripheral pulse regularity rather than beat-level cardiac activity.

Before pulse analysis, the PPG module evaluates signal quality. Recordings are checked for sufficient duration, finger coverage, pulse-peak content, physiologically plausible heart-rate values, periodicity, and peak prominence. The signal is resampled to 30 Hz, detrended, smoothed, normalized, and analyzed for pulse peaks. In the current implementation, recordings must contain at least 120 frames, last at least 10 s, and have at least 8 detected pulse peaks. The module also applies thresholds for heart-rate range, autocorrelation, red-channel dominance, peak prominence, and an aggregate quality score. Recordings that do not satisfy these criteria are excluded from further analysis.

For recordings that pass the quality checks, the application calculates inter-pulse interval variability measures, including the coefficient of variation and root mean square of successive differences (RMSSD). A pulse sequence is considered irregular when the coefficient of variation exceeds 0.10 or RMSSD exceeds 80 ms. The PPG module is therefore used to assess signal quality and pulse regularity following an ECG alert, rather than as an independent arrhythmia classification system.

The application maintains an on-device status log that includes information on sensor connectivity, activity state, ECG predictions, reconstruction training, PPG quality assessment, and pulse-regularity results. All logs are stored locally on the device and can be viewed through the application interface.

The Android implementation uses Java-based signal processing, model inference, and federated-learning components. Table 3 summarizes the main functional components of the application and their roles in the overall workflow. The deployed model package contains the trained network weights together with the metadata required for preprocessing, classification, local adaptation, and federated parameter exchange. The promoted Android model corresponds to the configuration obtained through PTB-XL masked-reconstruction pretraining followed by supervised MIT-BIH training and SVEB-aware calibration.

**Table 3:** Main functional components of the Android application.

Component	Function
Wearable ECG acquisition	Acquires real-time ECG signals from the Movesense sensor over Bluetooth Low Energy (BLE)
ECG preprocessing	Performs signal conditioning, beat-window validation, and preparation of ECG data for analysis
Beat representation	Detects R peaks and converts ECG segments into 68-dimensional ECG/RR feature vectors
On-device classification	Predicts normal (N), supraventricular ectopic (SVEB), and ventricular ectopic (VEB) beats from incoming ECG windows
Activity-aware gating	Restricts classification and model adaptation to static activity states identified through smartphone activity recognition
Alert management	Generates user notifications following abnormal ECG detections while applying confidence and rate-limiting rules
Federated adaptation	Performs reconstruction-based local learning and exchanges encoder/decoder parameters through the federated-learning framework
PPG assessment	Records smartphone-camera PPG signals and evaluates signal quality and pulse regularity following ECG alerts
Status logging	Stores local records of connectivity, ECG analysis, federated-learning events, and PPG assessment outcomes
User interface	Provides access to acquisition status, classification results, PPG assessments, notifications, logs, and user controls

## 4 Results

### 4.1 System realization and deployed mobile artifact

The proposed system is implemented as Cardio-FL, an Android application that combines wearable ECG acquisition, on-device arrhythmia classification, federated model adaptation, activity-aware operation, and smartphone-camera PPG assessment. ECG signals are streamed from a Movesense wearable sensor over Bluetooth Low Energy (BLE), processed locally on the smartphone, and converted into beat-level ECG/RR representations for classification. The application also supports PPG recording following ECG alerts, local status logging, and federated exchange of trainable model parameters.

Table 4 summarizes the main properties of the deployed system. The complete encoder-classifier-decoder model contains 5,575 parameters, of which 5,524 belong to the encoder and decoder and are included in federated learning. The classifier contains 51 parameters and remains fixed during on-device adaptation. This design enables representation learning from unlabeled ECG data while preserving the supervised classifier trained for N/SVEB/VEB beat classification.

**Table 4:** Summary of the deployed Cardio-FL system.

Characteristic	Value	Description
ECG source	Movesense ECG over BLE	Wearable ECG acquisition
Input representation	68-dimensional vector	ECG morphology and RR-interval features
Output classes	N, SVEB, VEB	ECG beat classification targets
Total model size	5,575 parameters	Complete deployed model
Federated-learning parameters	5,524 parameters	Updated during local and federated learning
Fixed classifier parameters	51 parameters	Excluded from adaptation
Federated payload	22,096 bytes	Parameters exchanged per communication round
Model bundle size	160 KB	Packaged model asset
Release APK size	15.2 MB	Installable Android application
Application implementation	24 Java classes	Acquisition, inference, adaptation, and user-interface components

To evaluate deployment feasibility, we measured several application-level runtime characteristics. These measurements are intended to assess implementation performance rather than clinical effectiveness. As summarized in Table 5, model inference required less than 1 ms per beat on average, while the complete preprocessing and inference pipeline required approximately 20 ms per beat. During a 30 min BLE streaming session, the application received 230,352 of 230,400 expected ECG samples, corresponding to an estimated sample loss of 0.02%, with no observed disconnects, connection failures, or parsing errors. Reconstruction-based local training completed in approximately 0.06 s for a batch of 32 ECG windows and three local training epochs. PPG signal-quality and pulse-regularity analysis required approximately 56 ms following acquisition of a 20–30 s recording.

**Table 5:** Runtime characteristics of the deployed Android application.

Metric	Evaluation setup	Result
ECG inference latency	100 ECG beat windows, repeated five times	0.64 ms/beat on average (range: 0.57–0.69 ms/beat)
ECG processing and inference latency	JSON parsing, preprocessing, and inference on 100 ECG windows, repeated five times	19.51±1.67 ms/beat
BLE streaming stability	30 min continuous ECG acquisition session	230,352 of 230,400 samples received (0.02% sample loss); no disconnects or processing errors
Local on-device training time	32 ECG windows, three local epochs, eight training rounds	0.058±0.019 s/round
PPG processing time	Analysis of a 20–30 s smartphone-camera PPG recording	55.6±18.4 ms
Battery consumption	30 min ECG acquisition and inference session in airplane mode	1% battery decrease (100%–99%)

Measurements are reported to assess implementation feasibility and resource requirements.

**Figure 5:**
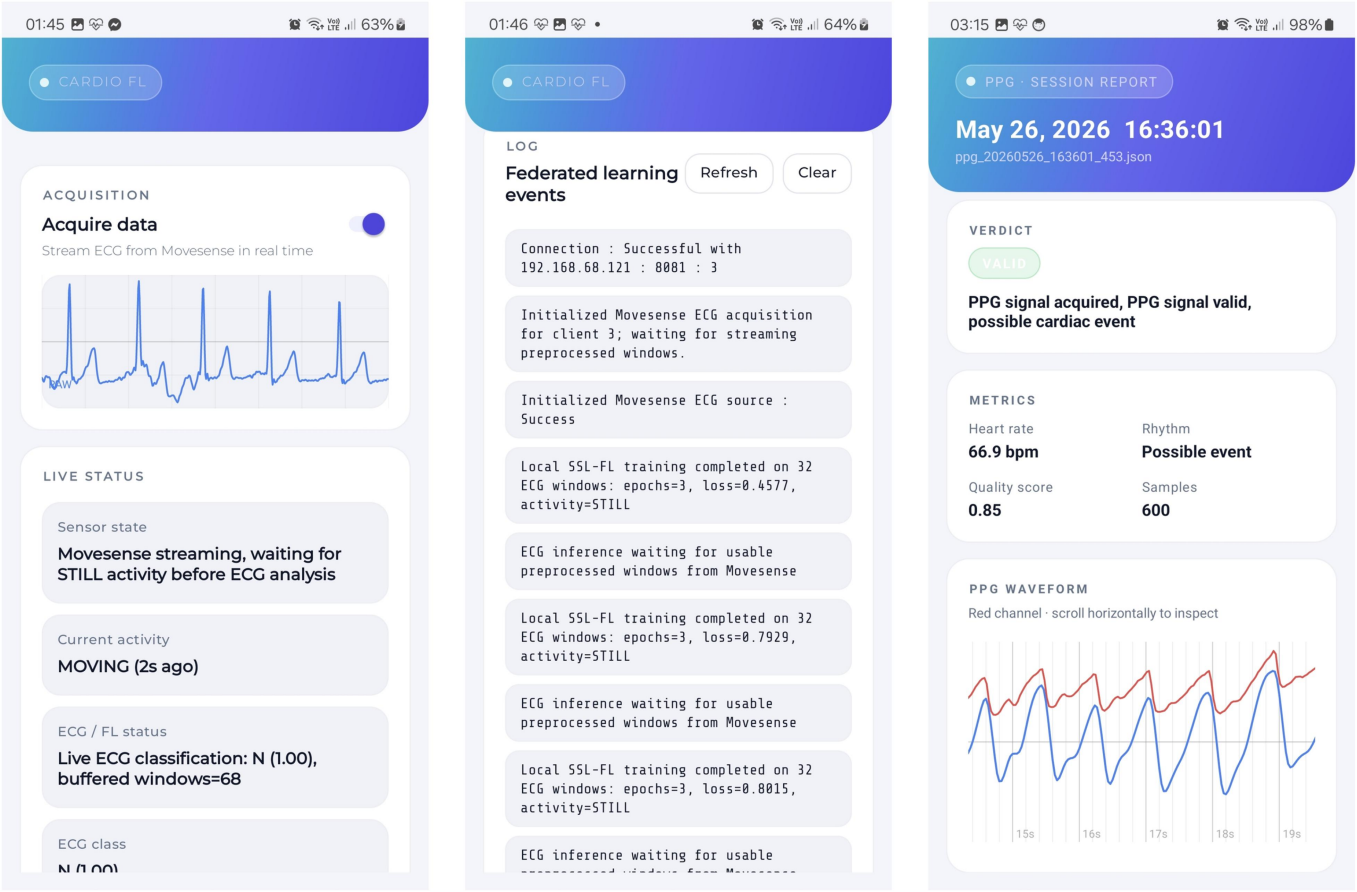
Representative screens from the Cardio-FL Android application. Left: ECG acquisition and beat-classification view showing sensor connectivity, activity state, and classification output. Center: federated-learning log displaying local training and communication events. Right: PPG assessment screen summarizing signal quality, pulse regularity, heart rate, and waveform information following a camera-based recording.

Figure 5 shows representative screens from the deployed Android application. The interface provides real-time information on ECG acquisition, activity state, classification results, federated learning events, and PPG assessment outcomes. Together with the runtime measurements in Table 5, these screenshots illustrate the integration of sensing, on-device inference, PPG assessment, and federated adaptation within a single mobile application.

### 4.2 Effect of masked ECG pretraining on deployed classification performance

We evaluated the effect of masked reconstruction pretraining on PTB-XL lead-II ECG data before supervised training on MIT-BIH beat classification. Table 6 compares the MIT-BIH-only baseline with several PTB-XL pretraining configurations. The final deployed model used three epochs of PTB-XL pretraining followed by supervised MIT-BIH training and SVEB-aware calibration.

**Table 6:** Effect of PTB-XL lead-II masked reconstruction pretraining and SVEB-aware calibration on the compact Android ECG model.

Training/decision configuration	Accuracy	Macro-F1	SVEB F1	VEB F1	Recon. MSE
MIT-BIH only, argmax	92.80%	69.24%	35.86%	75.44%	0.7243
PTB-XL, 1 epoch, argmax	93.18%	68.07%	29.38%	78.15%	0.7264
PTB-XL, 3 epochs, argmax	94.14%	72.52%	40.37%	79.98%	0.6847
PTB-XL, 3 epochs, SVEB-calibrated	**94.15%**	**74.53%**	**46.11%**	**80.31%**	0.6847
PTB-XL, 5 epochs, argmax	92.98%	67.09%	27.01%	77.74%	0.6975
PTB-XL, 10 epochs, argmax	91.69%	66.42%	32.24%	71.27%	0.6971
PTB-XL, 10 epochs ECG-only, argmax	91.64%	65.70%	30.75%	70.65%	0.6726

The final selected model uses three epochs of PTB-XL pretraining and validation-selected SVEB calibration.

Bold values indicate the highest classification performance across the reported metrics achieved by the selected deployed model configuration.

Compared with the MIT-BIH-only baseline, the selected model improved classification performance across all reported metrics. Accuracy increased from 92.80% to 94.15%, while macro-F1 increased from 69.24% to 74.53%. Performance improvements were also observed for both arrhythmia classes. SVEB F1 increased from 35.86% to 46.11%, and VEB F1 increased from 75.44% to 80.31%. Reconstruction mean squared error (MSE) decreased from 0.7243 to 0.6847.

**Figure 6:**
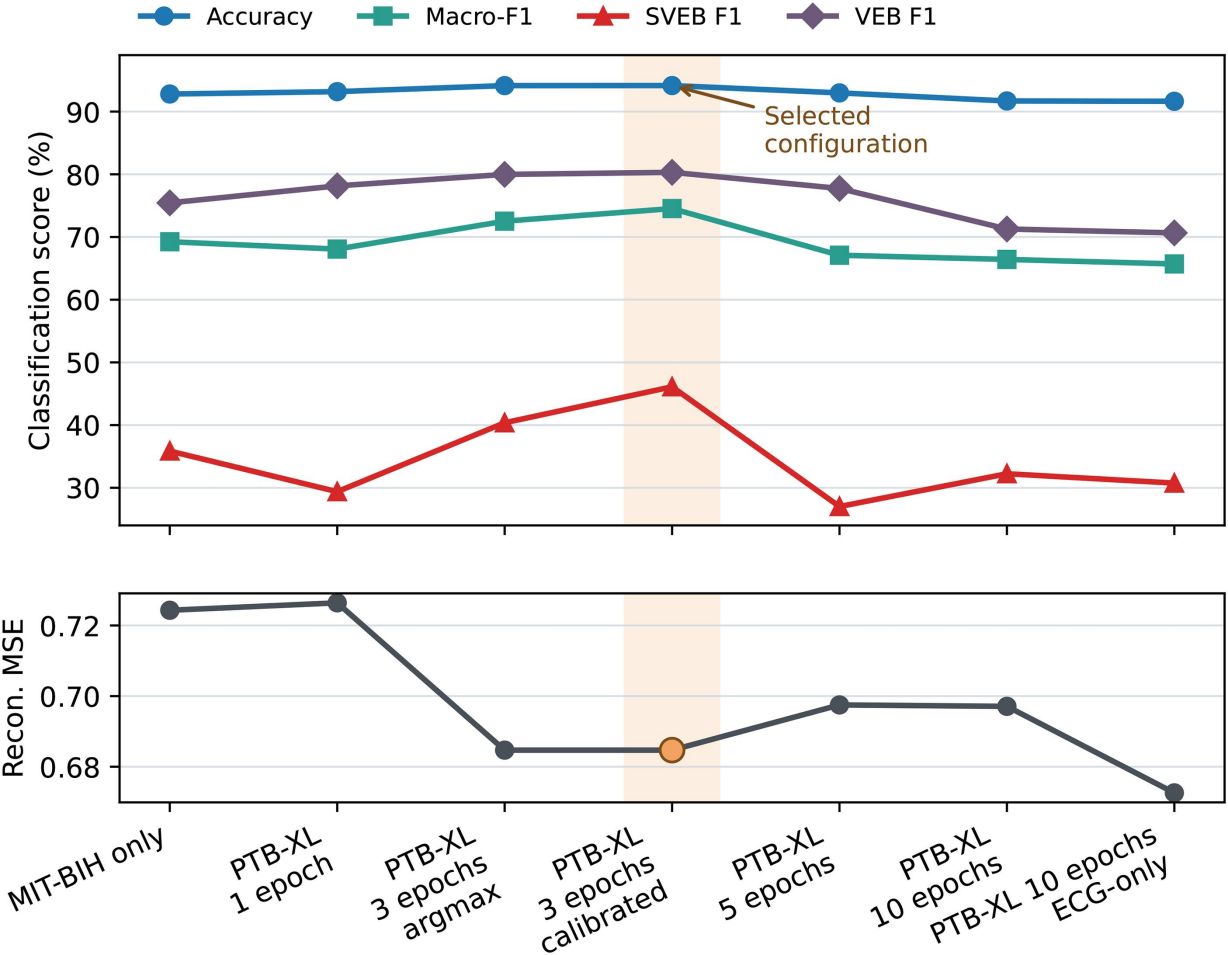
Effect of PTB-XL masked reconstruction pretraining on downstream MIT-BIH beat classification. The selected three-epoch configuration with SVEB-aware calibration provides the best joint trade-off across accuracy, macro-F1, SVEB F1, VEB F1, and reconstruction error.

The results indicate that limited PTB-XL pretraining improved downstream beat-classification performance. The best results were obtained after three pretraining epochs, whereas longer pretraining durations resulted in lower classification performance despite similar reconstruction error. Figure 6 illustrates this trend across all evaluated pretraining configurations. Among the evaluated configurations, the three-epoch model combined with SVEB-aware calibration achieved the highest accuracy, macro-F1, SVEB F1, and VEB F1 scores.

### 4.3 SVEB-aware calibration of the selected Android model

The selected ECG model initially used a standard argmax decision rule for N, SVEB, and VEB classification. Evaluation on the validation set showed that SVEB recall was substantially lower than that of the other classes, with many SVEB beats classified as normal. To improve SVEB detection, we evaluated a range of SVEB-specific probability thresholds and logit biases without modifying the model architecture or retraining the network.

The best validation-set performance was obtained using an SVEB probability threshold of $\tau_{\mathrm{SVEB}}=0.40$ together with an SVEB logit bias of 0.2. This calibrated operating point was subsequently evaluated on the held-out test set. As shown in Table 7, calibration improved macro-F1 from 72.52% to 74.53% and SVEB F1 from 40.37% to 46.11%, while overall accuracy remained unchanged. SVEB recall increased from 34.42% to 43.95%, and the proportion of SVEB beats classified as normal decreased from 47.50% to 41.45%.

**Table 7:** Effect of SVEB-aware calibration on classification performance.

Decision rule	Accuracy	Macro-F1	SVEB precision	SVEB recall	SVEB F1
Argmax baseline	94.14%	72.52%	48.80%	34.42%	40.37%
SVEB-calibrated	**94.15%**	**74.53%**	48.50%	**43.95%**	**46.11%**

Calibration parameters were selected on the validation set and evaluated on the held-out test set.

Bold values indicate the metrics that improved after applying the selected SVEB-aware calibration compared with the argmax baseline.

Table 8 summarizes the class-specific performance of the calibrated model. The highest performance was achieved for normal beats, with an F1-score of 97.17%, followed by VEBs with an F1-score of 80.31%. SVEB detection remained the most challenging task, although calibration improved both recall and F1-score relative to the uncalibrated model.

**Table 8:** Class-specific performance of the SVEB-calibrated ECG classification model.

Class	Precision	Recall	F1-score
N	97.67%	96.68%	97.17%
SVEB	48.50%	43.95%	46.11%
VEB	73.82%	88.04%	80.31%

To assess how stable the reported test-set performance was across MIT-BIH records, we applied record-level bootstrap resampling to the selected calibrated Android model, as described in Section 3.3. The resulting 95% confidence intervals were 90.32%–97.11% for accuracy, 59.35%–79.69% for macro-F1, 4.57%–59.60% for SVEB F1, and 55.73%–91.43% for VEB F1. The wide SVEB F1 interval reflects the small number and uneven distribution of SVEB beats across records, confirming that SVEB detection remains the main classification limitation of the compact Android model.

Furthermore, for the SVEB-aware calibration step, paired record-level bootstrap resampling was used to compare the calibrated decision rule with the argmax baseline. Compared with the argmax baseline, calibration changed macro-F1 by +2.01 percentage points (95% CI: −0.32 to +3.70), SVEB F1 by +5.74 percentage points (95% CI: −0.29 to +9.08), and SVEB recall by +9.53 percentage points (95% CI: +2.23 to +10.77). The clearest effect of calibration was therefore improved SVEB recall, while the macro-F1 and SVEB F1 improvements should be interpreted cautiously at the record level.

**Figure 7:**
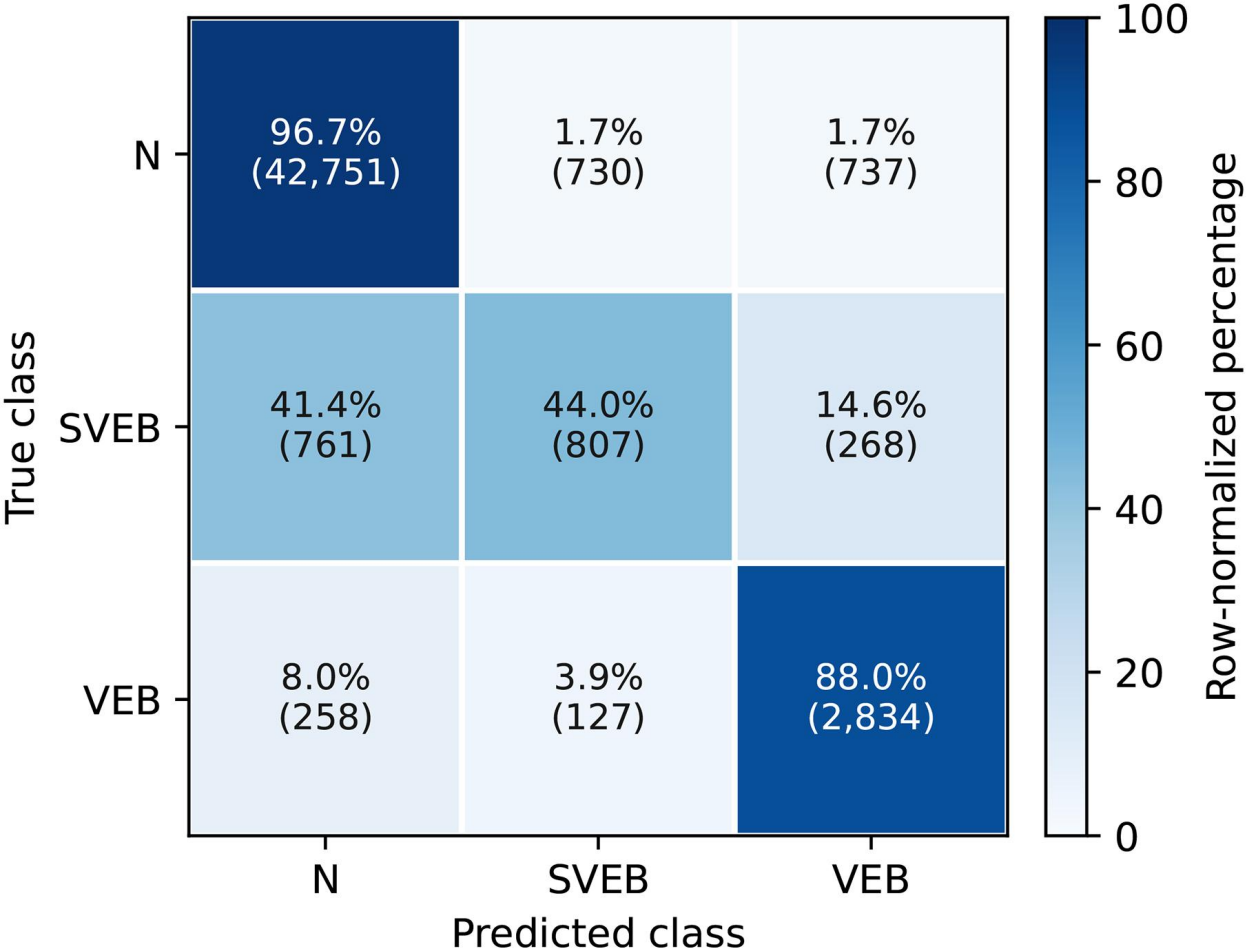
Normalized confusion matrix of the calibrated ECG classification model. Calibration primarily increased correct SVEB classification and reduced confusion between SVEB and normal beats.

The confusion matrix in Figure 7 illustrates the effect of calibration on class-level predictions. The largest change was observed for SVEB beats, for which correct classification increased from 34.42% to 43.95%. The proportion of normal beats classified as SVEB increased slightly from 1.30% to 1.65%, while VEB performance remained largely unchanged.

### 4.4 Feature contribution of the ECG/RR representation

To assess the contribution of the compact 68-dimensional ECG/RR representation, we performed a post hoc ablation analysis on the selected calibrated model. Without retraining the model, either the four RR-interval features or the 64 derivative ECG features were replaced with their corresponding training-set mean values, allowing us to assess the relative contribution of rhythm context and local ECG morphology.

Replacing the RR features reduced macro-F1 from 74.53% to 59.38% and eliminated SVEB detection, showing that timing context was essential for recognizing supraventricular ectopic beats. Replacing the derivative ECG features reduced macro-F1 to 47.26% and eliminated VEB detection, indicating that local morphology was essential for identifying ventricular ectopic beats. Table 9 summarizes the effect of each feature group on the classification metrics. Together, these results support the design choice of combining RR-based rhythm context with derivative ECG morphology in the proposed ECG/RR representation.

**Table 9:** Post hoc feature ablation of the selected calibrated ECG model.

Input setting	Accuracy	Macro-F1	SVEB F1	VEB F1
Full ECG/RR input	94.15%	74.53%	46.11%	80.31%
RR features replaced	93.73%	59.38%	0.00%	81.55%
ECG derivative replaced	90.42%	47.26%	44.37%	0.00%

Features were replaced by training-set means without retraining.

### 4.5 Comparison with a fixed-inference ECG classifier

To contextualize the performance of the deployed model, we compared it with an earlier ECG classifier developed during the project. This comparator followed a matched-filter-inspired design based on the ECG/RR representation proposed by Farag (1) and was optimized exclusively for fixed on-device inference. Unlike the deployed Cardio-FL model, it did not include an encoder-decoder architecture or support reconstruction-based local and federated adaptation.

Table 10 summarizes the comparison. The fixed-inference classifier achieved higher classification performance, reaching 95.78% accuracy, 85.47% macro-F1, 69.48% SVEB F1, and 89.19% VEB F1 on the MIT-BIH DS2 test split. In comparison, the deployed Android model achieved 94.15% accuracy, 74.53% macro-F1, 46.11% SVEB F1, and 80.31% VEB F1.

**Table 10:** Comparison between the deployed Cardio-FL model and an earlier fixed-inference ECG classifier.

Model	Accuracy	Macro-F1	SVEB F1	VEB F1	Deployment role
Selected calibrated Android model	94.15%	74.53%	46.11%	80.31%	Adaptive mobile model
Earlier fixed-inference cascade	95.78%	85.47%	69.48%	89.19%	Fixed-inference classifier

The comparator is included to provide context for classification performance and does not support reconstruction-based adaptation or federated learning.

The largest performance difference was observed for SVEB detection, where the fixed-inference classifier achieved substantially higher recall and F1-score. However, the two models were designed for different objectives. The fixed-inference classifier was optimized solely for classification performance, whereas the deployed Cardio-FL model incorporates reconstruction-based adaptation and federated learning in addition to beat classification. The comparison therefore provides a reference point for the current classification performance of the deployed system rather than a direct replacement candidate.

### 4.6 Wearable ECG acquisition and mobile workflow verification

To evaluate wearable ECG acquisition within the deployed system, pilot recordings were collected using the Movesense sensor under three activity conditions: still, walking, and jumping. These recordings were used to assess signal acquisition, data parsing, duration estimation, and sampling-rate consistency during operation of the Android application.

Table 11 summarizes the recording characteristics. Across all three conditions, the estimated sampling frequency remained close to the expected 200 Hz acquisition rate, with recording durations ranging from 124.8 s to 194.0 s. These results indicate stable ECG acquisition and processing across the tested activity conditions.

**Table 11:** Summary of pilot ECG recordings collected with the Movesense sensor under different activity conditions.

Condition	Samples	Duration (s)	Estimated sampling rate (Hz)
Still	36,465	182.315	200.01
Walking	38,801	193.995	200.01
Jumping	24,961	124.795	200.02

**Figure 8:**
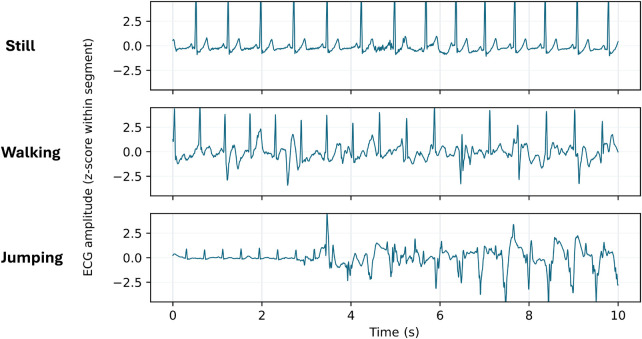
Representative ECG recordings acquired with the Movesense sensor during still, walking, and jumping activities. Increased motion produces visible signal disturbances, particularly during walking and jumping.

Figure 8 shows representative ECG segments recorded during the three activities. The recordings illustrate the influence of user motion on signal quality, with larger motion-related disturbances visible during walking and jumping than during stationary recording. These observations are consistent with the activity-aware gating strategy implemented in the Android application, which restricts inference and model adaptation to periods of low user activity.

### 4.7 Federated-learning integration

The deployed Android application supports federated reconstruction learning through the Flower framework. During local training, buffered unlabeled ECG windows are used to update the encoder and decoder using the reconstruction objective described in Section 3.4. The classifier remains fixed and is excluded from all local and federated updates. For testing, the federated learning configuration used 20 communication rounds, local batches of 32 ECG windows, and 3 local training epochs per round. The minimum number of participating clients was set to one to simplify the testing.

Table 12 summarizes the federated-learning characteristics of the current implementation. Raw ECG and PPG recordings remain on the device, while only encoder and decoder parameters are exchanged with the server. The federated payload consists of 22,096 bytes of model parameters before communication overhead, corresponding to the 5,524 trainable parameters of the encoder and decoder.

**Table 12:** Federated-learning characteristics of the deployed Android implementation.

Property	Implementation	Description
Federated framework	Flower	Client-server parameter exchange
Updated parameters	Encoder and decoder	Parameters included in federated updates
Fixed parameters	Classifier head	Excluded from federated updates
Local objective	Reconstruction loss	Training on unlabeled ECG windows
Raw data sharing	None	ECG and PPG recordings remain on device
Communication payload	22,096 bytes	Encoder and decoder parameters
Testing configuration	20 rounds, 1 client, batch size 32, 3 local epochs	System validation setup

These results demonstrate the successful integration of local reconstruction learning and federated parameter exchange in the Android application, as described in Section 3.4. The implemented workflow supports adaptation from unlabeled ECG data without transferring raw physiological recordings outside the device. We further evaluated this adaptation mechanism through a post hoc pseudo-client experiment on the held-out MIT-BIH test records, comparing fixed inference, local reconstruction adaptation, and federated reconstruction adaptation.

Each held-out MIT-BIH test record was treated as a simulated client. The first half of each record was used as unlabeled adaptation data, and the second half was used for labeled evaluation. As shown in Table 13, local and federated reconstruction adaptation reduced reconstruction error. However, the classification-performance changes were limited and not consistent across records. Relative to no adaptation, local adaptation changed macro-F1 by +0.27 percentage points (95% CI: −0.33 to +0.91), while federated adaptation changed macro-F1 by +0.04 percentage points (95% CI: −0.28 to +0.37). Since both intervals include zero, these results do not provide clear evidence of improved classification performance. The introduced reconstruction-based adaptation improved the reconstruction objective, but this did not translate into a clear classification-performance benefit in the current pseudo-client evaluation.

**Table 13:** Post hoc pseudo-client comparison of fixed inference, local reconstruction adaptation, and federated reconstruction adaptation.

Setting	Accuracy	Macro-F1	SVEB F1	VEB F1	Recon. MSE
No adaptation	93.94%	76.18%	51.60%	80.01%	–
Local reconstruction adaptation	94.14%	76.45%	51.51%	80.78%	0.6426→0.6001
Federated reconstruction adaptation	93.98%	76.22%	51.42%	80.29%	0.6376→0.6224

## 5 Discussion

This work extends arrhythmia monitoring beyond offline classification by integrating wearable sensing, on-device inference, multimodal confirmation, and federated adaptation within a single mobile system. Recent studies have demonstrated the feasibility of portable ECG monitoring, PPG-based rhythm screening, and privacy-preserving federated learning as separate research directions (4–8, 11, 12). Cardio-FL combines these components within a smartphone-based workflow in which wearable ECG serves as the primary rhythm signal, smartphone-camera PPG provides a secondary pulse assessment, activity recognition restricts operation to more reliable recording conditions, and federated learning enables adaptation from unlabeled on-device ECG data.

The main contribution of this work is the integration of these components into a single mobile monitoring workflow. Rather than focusing exclusively on classification performance, the system addresses several practical requirements of mobile cardiac monitoring, including wearable signal acquisition, local signal processing, resource-constrained inference, user feedback, privacy-preserving adaptation, and operation under changing user and environmental conditions. These considerations are important because the usefulness of mobile arrhythmia monitoring depends not only on classification accuracy but also on reliability, usability, privacy, and the ability to accommodate user-specific signal variability.

The pretraining results demonstrate the value of using large unlabeled ECG datasets to initialize compact mobile models. PTB-XL masked-reconstruction pretraining followed by supervised MIT-BIH training improved classification performance across all reported metrics, increasing accuracy from 92.80% to 94.15% and macro-F1 from 69.24% to 74.53%. Improvements were also observed for both arrhythmia classes, with SVEB F1 increasing from 35.86% to 46.11% and VEB F1 increasing from 75.44% to 80.31%. However, the pretraining analysis further showed that longer reconstruction pretraining did not lead to additional performance gains. These results suggest that masked reconstruction is most effective as an initialization strategy rather than a replacement for supervised beat-classification training.

This observation is consistent with the encoder–classifier–decoder design used throughout the system. Separating the trainable encoder and decoder from the fixed classifier allows the representation to adapt to local ECG characteristics while preserving the classifier trained on expert-annotated data. This separation is particularly relevant for mobile deployment, where large amounts of unlabeled ECG data may be available, but reliable beat annotations are generally not.

The comparison with the fixed-inference classifier highlights an important trade-off between classification performance and adaptability. The earlier classifier achieved substantially higher SVEB and macro-F1 performance, indicating that the classification component of the current model can be improved further. At the same time, the deployed Cardio-FL model offers capabilities not available in the fixed-inference approach, including reconstruction-based adaptation and federated learning from unlabeled ECG data. Future work should therefore focus on improving the classification head while maintaining the adaptation framework introduced in this study.

The SVEB results demonstrate the importance of decision-rule calibration for compact mobile classifiers. Under the standard argmax decision rule, many SVEB beats were classified as normal. Applying a validation-selected SVEB threshold and logit bias increased SVEB recall from 34.42% to 43.95% and SVEB F1 from 40.37% to 46.11% without reducing overall accuracy. Nevertheless, SVEB remained the most challenging class, indicating that future work should further strengthen both rhythm-context modeling and classifier design.

These findings indicate that the present compact model should not be interpreted as a standalone detector of supraventricular ectopy or as a clinically validated diagnostic classifier. The SVEB output should instead be considered an implementation-stage monitoring signal. Before such predictions can support clinical decision-making, SVEB sensitivity must be improved and validated prospectively under realistic monitoring conditions.

The feature-ablation analysis provides further insight into this limitation. Replacing the RR features with their training-set means reduced SVEB F1 to 0.00%, while VEB F1 remained high. Conversely, replacing derivative ECG morphology reduced VEB F1 to 0.00%, while SVEB F1 was partly preserved. This pattern supports the interpretation that SVEB detection depends strongly on rhythm-context information, since supraventricular ectopic beats may resemble normal beats in single-beat morphology. In contrast, VEB detection appears to rely more heavily on local ECG morphology, particularly QRS-shape information. Future model versions should therefore explore richer RR-context modeling or a staged classification strategy that separates morphology-driven VEB recognition from rhythm-context-driven SVEB recognition.

The smartphone-camera PPG module complements ECG-based monitoring by providing a supporting assessment of signal quality and pulse regularity after ECG alerts. ECG remains the primary signal because it directly measures cardiac electrical activity and supports beat-level analysis. PPG, in contrast, provides an indirect measure of peripheral pulse dynamics and is more susceptible to motion, contact, illumination, and perfusion artifacts. Used in this supporting role, PPG can enrich the user workflow by providing a second physiological observation when an ECG anomaly is detected.

The federated-learning component addresses a challenge that is often overlooked in benchmark-focused ECG studies: everyday use generates large volumes of unlabeled physiological data. Clinician annotations are rarely available during routine monitoring, while user-provided labels are typically sparse and unreliable. Reconstruction-based learning is therefore well-suited to the mobile setting because it can exploit unlabeled ECG windows collected during ordinary use. Keeping raw ECG recordings on the device also addresses important privacy considerations. At the same time, federated learning should not be viewed as a complete privacy solution. Model updates may still reveal information under certain threat models, and mechanisms such as secure aggregation or differential privacy may be required in higher-risk deployment scenarios (23, 24).

The pseudo-client adaptation experiment further clarifies the role of federated learning in the present study. Local and federated reconstruction updates reduced reconstruction error, indicating that the adaptation objective was optimized as intended. However, the corresponding macro-F1 changes relative to fixed inference were limited, and the paired record-level confidence intervals included zero. These findings show that the proposed workflow provides a privacy-preserving adaptation pathway, while further evaluation on genuinely heterogeneous users, sensors, and recording conditions is needed to determine whether reconstruction-based federated adaptation can improve arrhythmia classification.

Activity-aware gating represents another important design decision. Rather than processing all incoming ECG windows equally, the application restricts inference and model adaptation to static activity states. The pilot wearable recordings demonstrated that motion can substantially affect ECG signal quality, particularly during walking and jumping. Restricting operation to periods of lower activity reduces the likelihood that motion-contaminated recordings influence either arrhythmia detection or model adaptation. Future versions may incorporate explicit signal-quality assessment or motion-aware artifact detection, but the current approach favors simplicity and interpretability. This design choice prioritizes reliable signal windows, but it also reduces coverage outside static periods, so arrhythmic events occurring during physical activity may be missed or delayed. Future versions should therefore extend the current static-only strategy toward motion-aware signal-usability estimation, allowing the system to retain reliable ECG windows during activity while excluding segments affected by motion artifacts.

Several limitations should be acknowledged before clinical translation. The reported classification results are based on public ECG datasets and do not constitute prospective clinical validation. Although MIT-BIH and PTB-XL provide valuable resources for reproducible development, they do not fully capture the signal characteristics encountered in long-term mobile monitoring. Similarly, the pilot Movesense recordings were collected to verify wearable acquisition and processing under different activity conditions, rather than to demonstrate real-world diagnostic performance. The supporting modules should also be interpreted within the scope of the proposed prototype. The PPG module currently assesses signal quality and pulse regularity but is not a validated arrhythmia classification system. Likewise, the federated-learning implementation demonstrates on-device reconstruction learning and parameter exchange, but the pseudo-client analysis did not show a robust classification benefit from local or federated reconstruction adaptation. Finally, the Android prototype requires prospective evaluation under realistic deployment conditions. Pilot measurements of inference latency, BLE stability, local training time, and battery consumption were reported to establish technical feasibility, but these results do not constitute the systematic validation required for real-world use. Further evaluation is needed across communication overhead, thermal behavior, long-term battery impact, user interaction burden, and performance during extended operation in diverse usage conditions.

These limitations define a clear validation pathway. Future studies should evaluate the system prospectively using wearable ECG recordings collected from multiple users under realistic operating conditions. Particular attention should be given to signal quality, activity state, latency, energy consumption, and long-term usability. The federated-learning component should be compared with fixed inference, local-only adaptation, and centralized retraining approaches, while future model development should focus on improving SVEB detection without compromising the separation between adaptation and classification. Furthermore, the PPG workflow should be evaluated against reference measurements to determine when it provides useful pulse-regularity information and when low-quality recordings should be rejected.

Overall, the results demonstrate the feasibility of integrating wearable ECG acquisition, on-device inference, reconstruction-based adaptation, activity-aware gating, and smartphone-camera PPG assessment within a single mobile application. Although the system is not presented as a clinically validated diagnostic device, it provides a practical framework for investigating privacy-preserving and adaptive arrhythmia monitoring under real-world mobile deployment constraints.

## 6 Conclusion

This paper presents Cardio-FL, a smartphone-based cardiac-monitoring system that combines wearable ECG acquisition, on-device arrhythmia classification, federated model adaptation, activity-aware operation, and smartphone-camera PPG assessment within a single Android application. The work extends arrhythmia monitoring beyond offline classification by addressing practical aspects of mobile deployment, including local signal processing, privacy-preserving learning, and operation under real-world sensing conditions.

The proposed system uses a compact encoder–classifier–decoder model in which the classifier remains fixed while the encoder and decoder support reconstruction-based learning from unlabeled ECG data. Experimental results showed that masked-reconstruction pretraining on PTB-XL improved downstream MIT-BIH beat-classification performance, increasing accuracy from 92.80% to 94.15% and macro-F1 from 69.24% to 74.53%. SVEB-aware calibration further improved the operating point, highlighting the importance of both representation learning and decision-rule selection for compact mobile classifiers.

The study also demonstrates the integration of wearable ECG acquisition, on-device inference, reconstruction-based adaptation, PPG-assisted assessment, and federated parameter exchange within a functioning Android application. Runtime measurements and pilot wearable recordings confirmed the technical feasibility of the proposed workflow under mobile deployment conditions.

The system should be viewed as a mobile-health research platform rather than a clinically validated diagnostic device. Further work is required to evaluate performance prospectively across diverse users and recording conditions, establish whether reconstruction-based federated adaptation improves classification robustness under non-IID real-world data, strengthen SVEB detection, and validate the PPG workflow against clinical reference measurements.

Overall, the results demonstrate that wearable ECG sensing, self-supervised representation learning, and federated on-device adaptation can be integrated into a practical mobile monitoring system. This provides a foundation for future privacy-preserving and adaptive arrhythmia-monitoring solutions that operate directly on personal devices while accommodating the variability of real-world physiological data.

## Data Availability

The datasets presented in this study can be found in online repositories. The names of the repository/repositories and accession number(s) can be found below: https://gitlab.fri.uni-lj.si/lrk/cardio-fl.
